# HEAR4Health: a blueprint for making computer audition a staple of modern healthcare

**DOI:** 10.3389/fdgth.2023.1196079

**Published:** 2023-09-12

**Authors:** Andreas Triantafyllopoulos, Alexander Kathan, Alice Baird, Lukas Christ, Alexander Gebhard, Maurice Gerczuk, Vincent Karas, Tobias Hübner, Xin Jing, Shuo Liu, Adria Mallol-Ragolta, Manuel Milling, Sandra Ottl, Anastasia Semertzidou, Srividya Tirunellai Rajamani, Tianhao Yan, Zijiang Yang, Judith Dineley, Shahin Amiriparian, Katrin D. Bartl-Pokorny, Anton Batliner, Florian B. Pokorny, Björn W. Schuller

**Affiliations:** ^1^ EIHW – Chair of Embedded Intelligence for Healthcare and Wellbeing, University of Augsburg, Augsburg, Germany; ^2^ Division of Phoniatrics, Medical University of Graz, Graz, Austria; ^3^ Centre for Interdisciplinary Health Research, University of Augsburg, Augsburg, Germany; ^4^ GLAM – Group on Language, Audio, & Music, Imperial College London, London, United Kingdom

**Keywords:** computer audition, digital health, digital medicine, speech and language disorders, auscultation

## Abstract

Recent years have seen a rapid increase in digital medicine research in an attempt to transform traditional healthcare systems to their modern, intelligent, and versatile equivalents that are adequately equipped to tackle contemporary challenges. This has led to a wave of applications that utilise AI technologies; first and foremost in the fields of medical imaging, but also in the use of wearables and other intelligent sensors. In comparison, computer audition can be seen to be lagging behind, at least in terms of commercial interest. Yet, audition has long been a staple assistant for medical practitioners, with the stethoscope being the quintessential sign of doctors around the world. Transforming this traditional technology with the use of AI entails a set of unique challenges. We categorise the advances needed in four key pillars: Hear, corresponding to the cornerstone technologies needed to analyse auditory signals in real-life conditions; Earlier, for the advances needed in computational and data efficiency; Attentively, for accounting to individual differences and handling the longitudinal nature of medical data; and, finally, Responsibly, for ensuring compliance to the ethical standards accorded to the field of medicine. Thus, we provide an overview and perspective of HEAR4Health: the sketch of a modern, ubiquitous sensing system that can bring computer audition on par with other AI technologies in the strive for improved healthcare systems.

## 1. Introduction

Following the rapid advancements in artificial intelligence (AI), and in particular those related to deep learning (DL) (1), digital health applications making use of those technologies are accordingly on the rise. Most of them are focused on diagnosis: from computer vision techniques applied to digital imaging (2) to wearable devices monitoring a variety of signals (3, 4), AI tools are being increasingly used to provide medical practitioners with a more comprehensive view of their patients—a trend which has been accelerating in the aftermath of the COVID-19 pandemic (5). Computer audition complements this assortment of tools by providing access to the audio generated by a patient’s body. Most often, this corresponds to speech produced by the patients—sometimes natural, mostly prompted (6–8). However, there exists a plethora of auditory signals emanating from the human body, all of which are potential carriers of information relating to disease.

These biosignals can be analysed either through specialised instruments or, more interestingly, through the use of off-the-shelf microphones embedded in everyday devices, such as smartphones, which are already being widely used by healthcare professionals in their day-to-day jobs (9). As such, they are poised to be an indispensable tool to assist doctors in making better decisions and acquiring a more holistic understanding of their patients. While AI monitoring systems could, theoretically, be deployed as standalone applications and make decisions without supervision, we envision the components of our system to assist doctors in their decision making processes, rather than substitute them.

Acquiring auditory biosignals is the first, crucial step in a computer audition pipeline. Oftentimes, this must be done in noisy environments where audio engineers have little to no control, e.g., in a busy hospital room or the patient’s home. This results in noisy, uncurated signals which must be pre-processed in order to become usable, a process which is extremely laborious if done manually. Automating this process becomes the domain of the first of four outlined pillars, **(I) Hear**, which is responsible for denoising, segmenting, and altogether preparing the data for further processing by the downstream algorithms.

Those algorithms typically comprise learnable components, i.e., functions whose parameters are going to be learnt from the consumed data; in the current generation of computer audition systems, the backbone of those algorithms consists of DL models. These models, in turn, are typically very data “hungry,” and require an enormous amount of computational resources and experimentation to train successfully. However, in the case of healthcare applications, such data might not exist, either due to privacy regulations which prohibit their open circulation, or, as in the case of rare or novel diseases, simply because this data does not exist. Yet doctors, and subsequently the tools they use, are commonly required to operate in such low-data regimes. Therefore, it is imperative to make these algorithms operational **(II) Earlier** than what is currently possible; this can be done, for example, by transferring knowledge from domains where data is widely available to data-sparse healthcare applications.

The first two pillars are of a more “engineering” nature; the third one requires more theoretical advances. Statistical learning theory, which forms the foundation of DL, is based on the core assumption that data are *independent and identically distributed* (10). In the healthcare domain, this translates to the hypothesis that the population of training patients is representative of the entire population—an assumption that often does not hold in practice. Instead, patients come from different backgrounds and are typically organised in sub-populations. Oftentimes, the level of analysis reaches all the way down to the individual; in this case, every patient is considered “unique.” Furthermore, the larger upside of using AI in medicine lies in providing more fine-grained information in the form of longitudinal observations. Handling the need for individualised analysis with multiple observations over time requires algorithms to operate **(III) Attentively** to individual—often changing—needs.

The last pillar corresponds to the translation of the mandate enshrined in the Hippocratic oath to computer audition, and more generally AI: any developed technologies must be developed and be able to operate **(IV) Responsibly**. The responsibility lies with the developers and users of the technology and is targeted towards the patients who become its objects. This informs a set of guidelines and their accompanying technological innovations on how data needs to be sourced, how algorithms must meet certain fairness requirements, and, ultimately, on “doing good” for mankind.

Finally, we would be amiss not to mention the potential applications that can benefit from the introduction of computer audition in healthcare. This becomes the central component which permeates all aspects of the four pillars: they exist insofar as they serve the overarching goal of providing medical practitioners with novel tools that can help them understand, analyse, diagnose, and monitor their patients’ **Health**.

**Figure 1:**
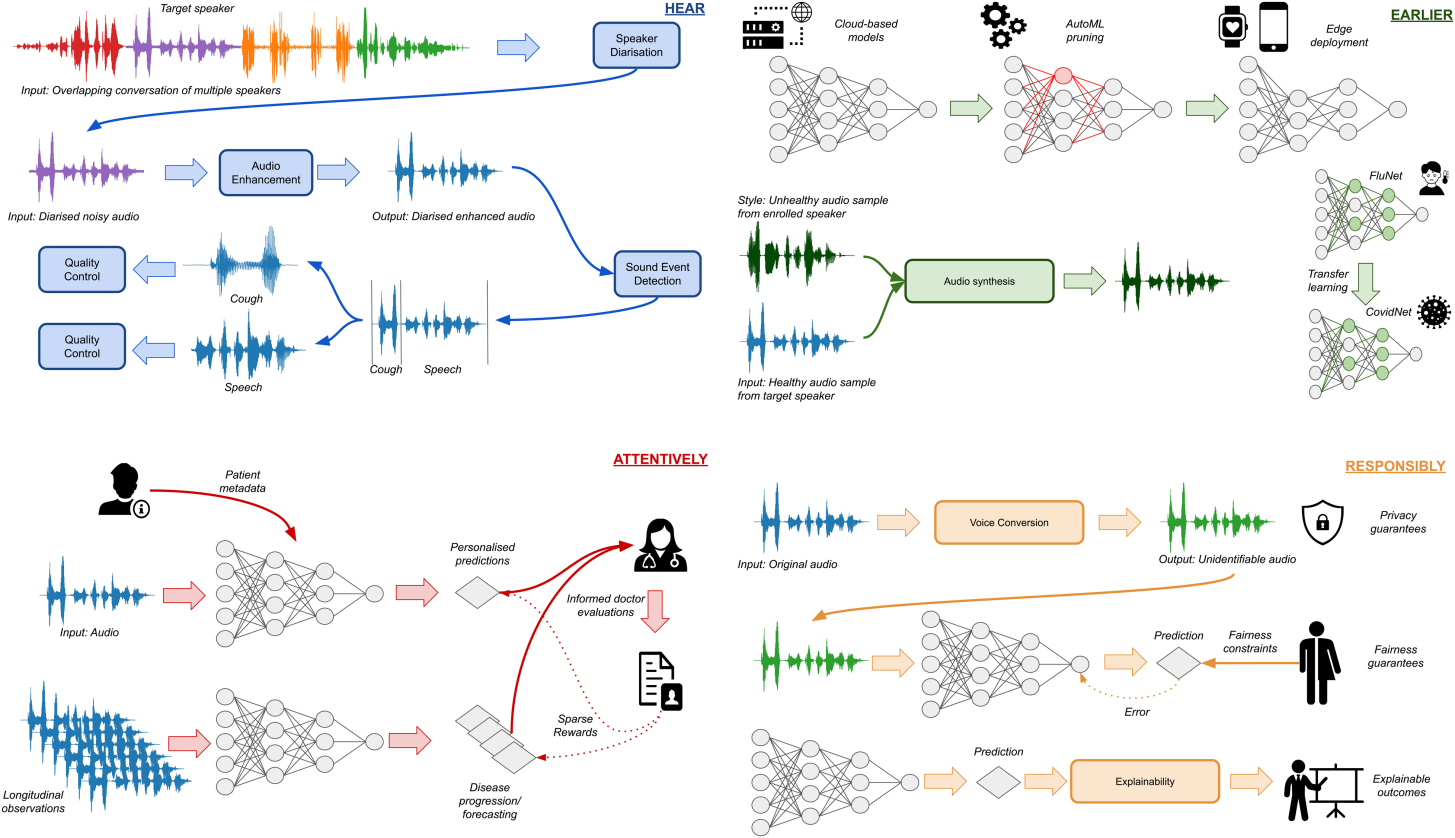
Overview of the four pillars for computer audition in healthcare. HEAR is tasked with capturing, identifying, and enhancing the target signal. EARLIER accounts for the need to deploy models at the edge to facilitate ubiquitous monitoring, as well as adapt to novel diseases using transfer learning and synthetic data generation. ATTENTIVELY incorporates personal information in the decision-making process, either using patient metadata or longitudinal observations, while assimilating doctor decisions in a human-in-the-loop framework. RESPONSIBLY ensures that developed models adhere to ethical requirements by safeguarding privacy, promoting fairness, and adding a layer of explainability.

An overview of the four pillars, as well as their interconnections are shown in Figure 1. In the following sections, we begin with an overview of the particular diseases in which we expect computer audition to make a decisive contribution, which lays the setting for the four pillars. We then proceed to analyse each of our four pillars in more detail and end with a discussion of how all four of them can be integrated in a practical architecture. Thus, we present **HEAR4Health**: an overview of recent advances and a blueprint for what needs to be done for audition to assume its rightful place in the toolkit of AI technologies that are rapidly revolutionising healthcare systems around the world.

Our work aims to go beyond existing surveys, which only concern themselves with the different technical aspects of computer audition, and link those aspects to the pragmatic requirements of the digital health setting. Therefore, instead of diving deep into technical details, we provide a broad, holistic coverage of the different components that are needed. Our work represents a roadmap and a blueprint for healthcare researchers and practitioners aiming to utilise computer audition to tackle a diverse set of challenges.

## 2. Healthcare applications

Naturally, any advances in computer audition targeted towards healthcare applications are inextricably tied to the specific medical conditions that lend themselves to modelling via audio; the necessary pre-requisite is that these conditions manifest themselves, at least to some extent, in auditory biomarkers emanating from the patients’ bodies. Historically, a significantly higher emphasis has been placed on vocalisations compared to other body acoustics such as heart sounds (6). Accordingly, this choice has shaped most of the existing approaches and, thus, also becomes the central point of our review. Table 1 shows the main ICD-11^1^ categories on which previous research has focused, as well as the specific diseases that previous studies have focused on and the auditory signals and symptoms used for acoustic disease monitoring and characterisation. In the following sections, we proceed to analyse each of those categories, presenting prior computer audition works that have focused on specific diseases, and discussing the impact that our HEAR4Health framework can have on them.

**Table 1:** Overview of diseases per ICD-11 category that are frequently investigated in the computer audition literature, as well as a list of auditory symptoms and signals used for their monitoring.

Disease group	Diseases	Symptoms	Relevant biosignals
Infectious or parasitic diseases	Tuberculosis	Coughing	Coughing
	Pertussis	Sore throat	Speech
	Influenza		
Mental, behavioural, or neurological disorders	Aphasia	Flatter tone	Speech
	Schizophrenia	Pauses	
	Autism	Strained articulation	
	Depression		
Sleep-wake disorders	Apnoeas	Snoring	Breathing
			Snoring
Diseases of the nervous system	Parkinson’s	Articulation problems	Speech
	Alzheimer’s	Phonation problems	Sustained vowels
	Multiple sclerosis		
	ALS		
	Cerebral palsy		
Diseases of the circulatory system	Arrythmias	Irregular heartbeat	Heartbeat
Diseases of the respiratory system	Asthma	Coughing	Breathing
	Bronchitis	Articulation problems	Coughing
	COPD	Phonation problems	Speech
	COVID-19		Sustained vowels
	Pneumonia		
Developmental anomalies	Angelman syndrome	Abnormal sounds	Baby sounds
	Fragile X syndrome	Abnormal speech	Speech
	Rett syndrome		

### 2.1. Infectious or parasitic diseases

This broad category covers several communicable diseases, from bacterial, gastrointestinal infections, to sexually transmitted diseases and viral infections, the majority of which do not manifest in auditory biomarkers; the ones that do, however, number several auditory symptoms such as (persistent) coughing or having a sore throat. The ones predominantly appearing in computer audition literature are: (respiratory) *tuberculosis* (1B10) (11–14); *pertussis* (1C12) (15, 16); and *influenza*(1E) (17). Existing works have predominantly focused on detecting and analysing coughs; in particular, the onset of DL and the increase in available data have unveiled the potential of detecting coughs and subsequently categorising them as pathological or not.

### 2.2. Mental, behavioural, or neurodevelopmental disorders

Disorders belonging to this category are described by the WHO as “syndromes characterised by clinically significant disturbance in an individual’s cognition, emotional regulation, or behaviour that reflects a dysfunction in the psychological, biological, or developmental processes that underlie mental and behavioural functioning.” The wide variety of symptoms of these diseases, often manifesting as speech and language pathologies, along with their widespread prevalence, have made them prime targets for the computer audition community (7, 18–20). Typical disorders are: (developmental) *aphasia* (6A01.20) (21, 22); *schizophrenia* (6A20) (23–25); *autism* (6A02) (26–30); *mood disorders* (6A60; 6A70), of which *depression* is the most commonly researched (31–33); and *anxiety or fear-related disorders* (6B) (34, 35). For example, aphasia has been linked to mispronunciation errors and increased effort (21, 22); schizophrenia manifests in slower response times in conversations (24); and blunted affect (23–25); depression results in a flatter tone (lower mean F0 values and range) with more pauses (32), and an increase in jitter and shimmer, indicative of more strained articulation.

### 2.3. Sleep-wake disorders

Research in sleep-wake disorders has been typically targeted to *breathing-disorders*—mainly apnoeas (36, 37), while some research has been focused on the detection of the resulting *sleepiness* (38). Apnoeas, on the one hand, mostly manifest as very loud snoring, which is caused by a prolonged obstruction of the airways and subsequent “explosive” inspirations. These signals can be automatically detected and analysed using auditory machine learning (ML) systems (39). Daytime sleepiness, on the other hand, has been mostly studied as a speech and language disorder; it manifests in lower speaking rates and irregular phonation (40).

### 2.4. Diseases of the nervous system

This family of diseases has adverse effects on memory, motor control, and cognitive performance. Its most widely studied sub-categories from a speech pathology perspective are Parkinson’s (8A00.0) (41, 42); Alzheimer’s (8A20) (43–45); multiple sclerosis (8A40) (46); amyotrophic lateral sclerosis (8B60) (47); and cerebral palsy (8D) (48). These manifest primarily in the speech signal, with dysarthria and dysphonia being the most common symptoms. For example, studies find that Parkinson’s shows up as increased roughness, breathiness and dysphonia, and higher F0 values (41, 42), Alzheimer’s results in more hesitation (43), multiple sclerosis leads to slower and more imprecise articulation, pitch and loudness instability, longer and more frequent pauses (46), and cerebral palsy shows up as dysarthria, hypernasality, and imprecise articulation of consonants (48).

### 2.5. Diseases of the circulatory system

Auscultation has been a mainstay of a medical examination since the invention of the stethoscope by Renë Laennec in 1816, by now a trademark of medical practitioners around the world (49). It is particularly useful when listening to the sounds of the heart or the lungs of a patient. Accordingly, its digital equivalent can be immensely useful in detecting pathologies of the circulatory system, such as arrhythmias or congenital heart diseases. Analysing those signals has become the topic of multiple PhysioNet challenges (50) was also featured in the 2018 version of the ComParE series (51), with computer audition systems being developed to detect and classify abnormal events (“murmurs”) in phonocardiograms (52, 53).

### 2.6. Diseases of the respiratory system

These diseases can be broadly taxonomised as being related to the upper or lower respiratory tract. Prominent examples are bronchitis (CA20) (16); chronic obstructive pulmonary disease (COPD; CA22) (54, 55); asthma (CA23) (56); pneumonia (CA40) (57); and COVID-19 (RA01; designated under “codes for special purposes” due to the pandemic emergency) (58, 59). By nature of their symptomatology, these diseases are prototypical examples of ones that manifest in auditory biomarkers. Thus, different signals have been used to detect their presence, such as speech (60), breathing and coughing (61, 62), or sustained vowels (63). The most exemplary of those is COVID-19, whose devastating impact was felt around the world since its emergence in late 2019 and has led to a wave of renewed interest in computer audition for healthcare applications. In general, these diseases lead to more coughs, irregular breathing, phonation and articulation, and constrained airflow resulting in less loud and more strained vocalisations.

### 2.7. Developmental anomalies

Developmental disorders, such as the Angelman syndrome (LD90.0), Rett syndrome (LD90.4), and fragile X syndrome (LD55) manifest in divergent vocalisation and speech development patterns from an early age (29, 64–66). Infants with specific developmental disorders produce abnormal cooing sounds and less person-directed vocalisations, and their vocalisations are found to be of lower complexity as compared to typically developing infants. From a signal perspective, these anomalies manifest in speech, first in pre-linguistic sounds and later on in linguistic vocalisations of young children. As the emphasis is on children and young adults, they present an additional challenge to data collection, on the one hand due to ethical and privacy reasons, and on the other due to a potentially reduced compliance of children with recording requirements.

## 3. Hear

A cornerstone of computer audition applications for healthcare is the ability to *Hear*: that is, the set of steps required to capture and pre-process audio waves and transform them into a clear, useful, and high-quality signal. This is all the more true in the healthcare domain, where recordings are often made in hospital rooms bustling with activity or conducted at home by the non-expert users themselves. Therefore, the first fundamental step in an application is to extract only the necessary components of a waveform.

In general, this falls under a category of problems commonly referred to as *source separation and diarisation* (67, 68): the separation part corresponds to the extraction of a signal coming from a particular source amongst a mixture of potentially overlapping sources, whereas diarisation corresponds to the identification of temporal start and end times of components assigned to specific subjects. In healthcare applications, these target components are the relevant sounds; this can include vocalisations (both verbal and non-verbal) but also other bodily sounds that can be captured by specialised auditory sensors attached to their body, or general ones that are monitoring the environment. These sounds need to be separated from all other sources; these may include a medical practitioner’s own body sounds (e.g., their voice in doctor-patient conversations) or background environmental noise (e.g., babble noise in a hospital). Accordingly, successful preparation entails a) the ability to recognise which sounds belong to the target subject, b) the ability to detect their precise start and end times, and c) the ability to remove all other signals that co-occur during that time from the waveform.

Traditionally, these steps are tackled by specialised pipelines, which include learnable components that are optimised in supervised fashion (68). For example, the ability to recognise which sounds belong to the target subject is generally referred to as *speaker identification* (69). While this term is usually reserved for applications where speech is the sound of interest, it can also be generalised to other bodily sounds (70). Similarly, separation is typically done in a supervised way (68). During the training phase, clean audio signals are mixed with different noises, and a network is trained to predict the original, clean signal from the noisy mixture. As generalisability to new types of noise sources is a necessary pre-requisite, researchers often experiment with test-time adaptation methods, which adaptively configure a separation model to a particular source (71).

The crucial role of the *Hear* pillar becomes evident when considering data collection. There are three main data collection paradigms employed in healthcare applications: (a) the (semi-)structured doctor-patient interview, (b) ecological momentary assessments (EMAs) based on prompts (72), and, (c) passive, continual monitoring (73). All of them require very robust patient identification and diarisation capabilities. However, each comes with its own set of unique challenges that can be tackled by the *Hear* pillar. Structured interviews are often conducted in relatively quiet environments (e.g. a doctor’s office or laboratory); the challenge mainly relies in the use of far-field microphones that make the processing more complicated (e.g. resulting in reverberation) (74). The need for a robust *Hear* pillar is punctuated by the fact that response rates and speaking times during interviews are often very informative features for these types of diseases; their accurate estimation is only possible following a reliable diarisation step.

EMAs further complicate processing as they may take place in different environments, not necessarily quiet ones, as the patient can choose to conduct them in any environment of their choosing. Thus, denoising becomes a crucial factor for removing the unwanted interference from background noise. Passive monitoring represents the most challenging form of data collection. The auditory signals are potentially embedded in several, high-varying sources; detection then becomes the first crucial step. Voice activity detection is more mature than the detection of other types of bodily acoustics; still, even that suffers from robustness issues and is often a crucial bottleneck for successful applications (75). This is followed by a source separation step which attempts to extract the useful signals from any other sources, a feat which becomes more challenging for non-speech signals such as coughing, as this requires general source separation. For such symptoms, a major contribution of the *Hear* pillar would be to improve the detection of coughs in naturalistic environments; this would pave the way for continuous monitoring using smart wearables or smartphones to monitor (prospective) patients over time and detect a change in their frequency of their coughing over time.

Moreover, the audio processing can be formulated as a single, unified task of target audio extraction. The gold standard for digital health applications is not defined by human listening studies as in traditional source separation, but rather from the performance of downstream processing modules, with the goal being to increase their performance and robustness to noise. Overall, the aim of pre-processing is to reduce the uncertainty in real-life recordings by adapting to different environmental situations. Hence, it helps to provide a more robust interface that enables digital health applications. Finally, some techniques based on speech enhancement and source separation, such as signal-to-noise ratio (SNR) estimation, can be used to make a decision on whether a specific audio signal is suitable for further audio-based medical diagnosis, depending on the quality of the original recording and the processed audio.

## 4. Earlier

The major promise of digital health applications is their ubiquitous presence, allowing for a much more fine-grained monitoring of patients than was possible in the past. This requires the systems to work on mobile devices in an energy-efficient way. Additionally, these systems must be versatile, and easy to update in the case of new diseases, such as COVID-19. This requires them to generalise well while being trained on very scarce data. However, training state-of-the-art DL models is a non-trivial process, in many cases requiring weeks or even months, and is furthermore notoriously data intensive. Moreover, the technology required, such as high-end GPUs, is often expensive and has exceptionally high energy consumption (76).

There have consequently been increasing efforts to develop AutoML approaches that optimise a large network until it is executable on a low-resource device (77, 78). Many of these approaches focus on reducing the memory footprint and the computational complexity of a network while preserving its accuracy. These techniques have shown promise across a range of different learning tasks, however, their potential has not yet been realised for audio-based digital health applications.

On the issue of data efficiency, there has been a lot of research on utilising transfer learning techniques for increasing performance and decreasing the required amount of data. This is usually done by transferring knowledge from other tasks (79, 80), or even other modalities (81, 82). However, in the case of audio in particular, an extra challenge is presented by the mismatch between the pre-training and downstream domains (83). Recently, large models pre-trained in self-supervised fashion have reached exceptional performance on a variety of different downstream tasks, including the modelling of respiratory diseases (54), while showing more desirable robustness and fairness properties (84).

The implementation details of the *Earlier* pillar largely depend on the biomarkers related to the specific medical condition of interest. For example, in terms of mental disorders, which mostly manifest as pathologies of speech and language, it is mostly tied to generalisation across different languages. On the one hand, linguistic content itself is a crucial biomarker; on the other hand, it serves to constrain the function of acoustic features; thus, there is a need to learn multi-lingual representations that translate well to low-resource languages. For diseases manifesting in sounds other than speech signals, the *Earlier* pillar would then improve the data efficiency of their categorisation. For example, contrary to speech signals, for which large, pre-trained models are readily available (85), there is a lack of similar models trained on cough data; a lack partially attributable to the dearth of available data. This can be overcome, on the one hand, through the use of semi-supervised methods that crawl data from public sources (86), and, on the other hand, by pursuing (deep) representation learning methods tailored to cough sound characteristics.

When COVID-19 took the world by storm in early 2020, it represented a new, previously unseen threat for which no data was available. However, COVID-19 is “merely” a coronavirus targeting the upper and lower respiratory tracts, thus sharing common characteristics with other diseases in the same family (87). Transferring prior knowledge from those diseases, while rapidly adapting to the individual characteristics of COVID-19, can be another crucial factor when deploying auditory screening tools in the face of a pandemic.

Nevertheless, even after using transfer learning techniques, the problem of data sparsity still remains. In the audio domain, acquisition of data representative of the variety of signals seen at population level is time-consuming, costly and inefficient. A potential remedy could be found in *generating* new data. Many state-of-the-art, high-fidelity approaches for generating audio computationally are being developed, and these could be used to facilitate targeted data generation for handling underrepresented diseases. Co-opting these approaches for the digital health domain to generate (personalised) utterances of pathological speech and use them to augment the training data holds a lot of promise for mitigating the sparsity issue.

## 5. Attentively

Most contemporary digital health applications focus on the identification of subject states in a static setting, where it is assumed that subjects belong to a certain category or have an attribute in a certain range. However, many conditions have symptoms that manifest gradually (88), which makes their detection and monitoring over time a key proposition for future digital health applications. Furthermore, disease emergence and progression over time can vary between individuals (89–91). For example, the age at onset and the progression rate of age-related cognitive decline varies between individuals (89), while there is substantial heterogeneity in the manifestation and development of (chronic) cough across different patients (92). Focusing on these aspects of digital health by adapting to changes in distributions and developing personalised approaches can drastically improve performance.

Recent deep neural network (DNN)-based methods for personalised ML (30) and speaker adaptation (93) already pave the way for creating individualised models for different patients. However, these methods are still in their nascent stage in healthcare (94). Personalised ML is a paradigm which attempts to jointly learn from data coming from several individuals while accounting for differences between them. Advancing this paradigm for speech in digital health by utilising longitudinal data from several patients for learning to track changes in vocal and overall behaviour over time is a necessary precondition for the digital health systems of the future. This means that time-dependent, individualised distributions are taken into account for each patient, by that requiring the development of novel techniques better suited to the nature of this problem; in particular, developing versatile DL architectures consisting of global components that jointly learn from all subjects, and specialised ones which adapt to particular patients (95, 96). This novel framing will also enable faster adaptation to new patients by introducing and adapting new models for those patients alone.

On the other hand, speaker adaptation corresponds to disentangling speaker effects from biomarkers related to specific speaker states. This will be achieved by breaking down the input audio signal to a set of independent factors, enabling factors unrelated to the task at hand to be disregarded, such as speaker characteristics or speaker traits. The novel framework of causal representation learning (97), where deep neural networks are trained to disentangle independent factors, has yet to be utilised in the healthcare domain. Accordingly, DL architectures must be developed that can utilise this implicit factorisation to differentiate between speaker-specific factors and disease biomarkers.

Finally, a major proposition of computer audition is to supplement clinical evaluations and doctor visits, which are resource-intensive procedures, with cost-efficient AI-driven measurements, remotely collected in advance of the appointment with finer temporal resolution. In addition to potential research applications, in clinical practice, this detailed, objective record will provide insights to clinicians and enable more timely diagnostic investigations; for example, by using change point detection to identify changes in the patient’s state. The feedback from expert examinations, which is collected in more infrequent intervals, can then be incorporated using reinforcement learning. Reinforcement learning remains underutilised in the audio domain, largely because of the lack of an interactive environment where sparse rewards are available. However, digital health applications are ripe with sparse signals from medical practitioners in conjunction with asynchronous audio recordings that can be used to actively learn from sequences of observations in a constantly changing environment. Some of the audio recordings are collected in regular intervals, e.g. using smartphone apps or phone prompts, and are only supplemented by self-report measures when appropriate (96). However, such measures can only serve as a proxy to the target at hand; clinical evaluations, sometimes including specialised tests, are the gold standard in health state assessment. These more costly interventions are carried out infrequently compared to the remotely collected data, based on the decisions of medical practitioners. The asynchronous relationship between data recordings and targets present a fundamental problem for digital health applications. Tackling this challenge will become possible by developing a reinforcement learning framework for audio, where patient recordings will constitute the observations and clinical evaluations the “reward” from which the model will learn.

Adapting to individual characteristics is also of paramount importance. The *Attentively* pillar can become a cornerstone of future applications for monitoring mental health. Applications are already re-orienting towards longitudinal monitoring; this serves to provide more insight to a patient’s mental state over time, and lends itself well to personalised modelling. This will additionally help elucidate differences within this family of diseases; as discussed above, symptoms are often similar across different mood disorders, making their differentiation difficult. This obstacle can be overcome by contextualising a model to individual characteristics, such as patient histories or demographics, resulting in a hybrid AI system, comprising both data-driven and knowledge-based components. To the best of our knowledge this has not been utilised in computational research for digital health—presenting a prime opportunity for the *Attentively* pillar.

Naturally, the requirement for personal data raises serious technical and ethical challenges. Firstly, this information might not be available to the same extent for each patient. Furthermore, there is an explicit trade-off between personalisation and privacy; the more individual-level that is needed by a system, the more privacy-infringing it becomes. It is therefore necessary that any personalisation methods are optional; their use should be turned on or off depending on whether the data is available and the patient agrees to its use. Most methods already support this level of controllability as they can be trained in a multi-condition scenario with different combinations of available/missing data. By selectively dropping out personal information during training, the system can learn to generalise in situations where this information is not available during deployment.

## 6. Responsibly

The development of responsible digital health technology is a key pillar of future healthcare applications. This ensures trustworthiness and encourages the adherence of users to monitoring protocols. Consequently, addressing crucial factors and technology-related consequences in automated disease detection concerning human subjects in a real-world context is of paramount importance (98).

This pillar intersects with all previous ones and informs their design, adhering to an ‘ethical-by-design’ principle which is fundamental for healthcare applications. Naturally, a first requirement that applies to all pillars is one of evaluation: all components of a healthcare application need to be comprehensively evaluated with respect to all sub-populations and sensitive attributes. This holds true for all components of a computer audition system: from extracting the target audio signal (Hear) to generating efficient representations (Earlier) and adapting to individual characteristics (Attentively), any developed methods should perform equally for different sub-populations. The evaluation could be complemented by explainability methods, which explicitly search for biases in model decisions (99).

Aside from comprehensively evaluating all methods with respect to fairness, explicit steps must be taken to improve on those (100). To this end, adversarial (101) and constraint-based methods (102) have been proposed to learn fair representations. In adversarial debiasing, the main predictive network learns to perform its task while an adversary pushes it toward representations which obfuscate the protected characteristics. Constraint-based methods instead solve the main prediction task subject to fairness constraints (such as equality of opportunity); these methods rely on convex relaxation or game-theoretic optimisation to efficiently optimise the constrained loss function.

The second requirement placed on the three other pillars is privacy. For example, the *Hear* pillar could be co-opted to remove private information (e.g., via using keyword spotting to remove sensitive linguistic information). The *Earlier* pillar would then take the extracted signal and remove any paralinguistic information unrelated to the task; this could be achieved by targeted voice conversion that preserves any required signal characteristics but changes the patient’s voice to be unrecognisable (103).

A popular method to protect the privacy of an individual when analysing and releasing data is differential privacy (DP) (104). DP tries to prevent “attackers” from being able to determine whether a certain individual is included in the dataset or not, i.e., the contribution of an individual in the dataset to the data or query output is obscured (105). This is achieved by adding controlled noise or randomness to the data or query results. In this connection, a parameter ϵ is applied to determine the strength of protection provided by a differential privacy mechanism, at which smaller values of ϵ may lead to better privacy but less data utility. Therefore, the non-trivial choice of ϵ depends on the specific privacy requirements, risk tolerance, and the data sensitivity in order to best deal with this tradeoff between privacy and utility. However, the personal information embedded in audio signals is not needed for a successful prediction and can be removed prior to storing the data thus safeguarding the privacy of individual patients irrespective of failsafe mechanisms that protect the collected datasets, which may prove insufficient against future, more competent attackers.

Satisfying this requirement, however, is particularly challenging for the *Attentively* pillar, as there is a natural privacy-personalisation trade-off: the more private information is removed, the less context remains to be utilised for the target patient. The main solution to this obstacle is the use of federated learning (106): to ensure that sensitive information cannot be derived from central models, differential privacy methods have been proposed, such as differentially private stochastic gradient descent (107) and a private aggregation of teacher ensembles (108). These methods would update the global model backbone discussed in Section V, which is shared among all “clients,” while any personalised components would remain local—and thus under the protection of safety mechanisms implemented by the client institutions.

Finally, researchers need to focus on intersections between the investigated technology, the healthcare professional, and the patient. Understanding how and why a particular decision was made is critical for all stakeholders in the medical ecosystem. First and foremost, patients are entitled to an explanation for a particular diagnosis or proposed treatment plan (109, 110). These decisions will ultimately be made by doctors who utilise AI models as tools, and thus, they need to understand the outputs and workings of those models themselves. Finally, model developers can benefit from a better understanding of how their model works in order to improve it in future iterations. Explainable AI (XAI) provides a clear understanding of how an algorithm works and why it makes specific decisions. This information helps medical professionals trust and interpret the AI’s outputs, and it also makes it easier for them to explain the AI’s decisions to their patients. Expectedly, the community has recently engaged in substantial research efforts to mitigate this problem, leading to a wave of novel XAI techniques (111–114).

XAI methods can be broadly categorised into two main categories: model-based (global) and instance-based (local) (115). Model-based methods, such as surrogate models or layer visualisation techniques, attempt to understand the inner mechanisms of a particular model. These methods give an understanding of how a model makes decisions over multiple instances. In contrast, instance-based methods focus on why a particular decision was made for each particular instance. These methods attempt to attribute the decision to the characteristics of that specific instance. Finally, a particularly pertinent explainability method for healthcare applications is counterfactional explainability (116), which provide a natural interface for doctors to evaluate alternative outcomes through the language of counterfactuals (“what would the decision have been if feature X had a different value?”). Ultimately, the goal of a comprehensive system should be to combine an assortment of different XAI methods and provide a well-rounded understanding of how auditory models work and why they make specific decisions.

In addition to these by now established XAI methods, recent advances in generative AI have paved the way for a more natural presentation of explanations to the end-user. For instance, the recent success in mapping different modalities to text by aligning the learnt representation spaces of large multimodal foundation models has enabled the provision of *textual explanations*, which can be seen as a form of captioning (117), with the difference that the must conform to XAI requirements (i.e., fidelity and correctness). For auditory models in particular, a more natural way to present explanations would be the *sonification* of explanatory information (118). In a nutshell, sonification entails the generation of audio that conveys information in an easily digestible way. At a basic level, this might simply correspond to identifying those constituents of an auditory signal which were most relevant for a particular decision and playing them back to the user (a method often used in biofeedback for training (119)), though with the advent of generative audio models, more advanced explanations will become possible.

Finally, like *Earlier*, low-resource languages become the subject of the *Responsibly* pillar as well; the majority of studies has been performed on English data, due to their wider availability. However, due to the widespread nature of mood disorders, it is imperative to extend the applicability of computer audition algorithms to a wider gamut of languages (and cultures). There is an equal lack of work on fairness aspects relating to cough detection and categorisation; in particular, we expect age to play a crucial work both in the frequency, and the acoustic properties of cough signals; this would fall under the auspices of the *Responsibly* pillar, which should be tasked first with understanding, and subsequently mitigating, differences in performance across different populations. Similar to explainability, these aspects of fairness could be embedded in a counterfactual framework (120) which would allow medical practitioners to examine alternative scenarios for algorithmic predictions (“what would the decision be if the patient was female instead of male?”).

## 7. HEAR4Health: a blueprint for future auditory digital health

Early diagnosis, ideally even before symptoms become obvious to individuals in their daily lives, allows very early interventions, maximising the likelihood of successful treatments and a positive outcome, and optimising public health expenditures. While early diagnosis will not enable a curative treatment of all diseases in all cases, it provides the greatest chance of preventing irreversible pathological changes in the organ, skeletal, or nervous system, as well as reducing chronic pain and psychological stress. In some cases, early intervention can prevent the emergence of related long-term consequences. From a public health perspective, early detection is also an effective way to minimise the spread of contagious diseases—as became evident during the COVID-19 pandemic.

Audio signals are well suited to such a non-invasive early diagnosis strategy, as they can be easily acquired anywhere and anytime using ubiquitous smart devices. A key differentiating factor of audition, as opposed to other modalities, is the nature of the signals that are used for monitoring patients. This can be audio recordings of the voice (e.g. sustained vowels, social interactions and interviews), body sounds (e.g. heartbeat, breathing, coughing, and snoring sounds), and audio recordings of an individual’s acoustic environment (e.g. extracting information about frequency of communicative acts and emotional states during interactions, or noise exposure) with the aim of developing tools and methods to support the earlier diagnosis of acute and chronic diseases. The nature of those signals presents new challenges, and new opportunities, for future healthcare systems. In the present section, we attempt to sketch out a blueprint for bringing existing and upcoming advances of computer audition technologies out of the lab and into the real world of contemporary medicine.

Unifying the four pillars results in a working digital health system which we name *HEAR*. Our system can be used to supplement the decision-making of practitioners across a wide facet of diseases. In general, we anticipate two distinct functioning modes for it. On the one hand, it can be used as a general-purpose *screening* tool to monitor healthy individuals and provide early warning signs of a potential disease. This hearing with “all ears open” mode takes a holistic approach, and emphasises a wide coverage of symptoms and diseases, thus functioning as an early alarm system that triggers a follow-up investigation. Following that, it can be utilised to *monitor* the state of patients after they have been diagnosed with a disease, or for measuring the effect of an intervention. This second, more constrained setting necessitates a ‘human-in-the-loop’ paradigm, where the doctor isolates a narrower set of biomarkers for the system to monitor—now with more focus and prior information about the patient’s state—which is then reported back for each new follow-up. Through this loop, HEAR provides vital information of a high-resolution temporal scale, thus facilitating more personalised interventions and helping strengthen the doctor-patient link.

A key enabling factor for both operating modes will be the co-opting of ubiquitous auditory sensors as medical screening devices: the most obvious candidates would be smartphones, but also other IoT devices with audio recording capabilities, such as smartwatches. These would rely on active or passing auditory monitoring to identify potential symptoms. In the case of passive monitoring, the onus would be on the *Hear* pillar to detect them: in the case of speech, utilising voice activity detection, identification, diarisation, and separation to extract the target voice, while additionally performing audio event detection to detect coughs, sneezes, snores, or other bodily sounds. Analysing the frequency of symptoms (in the case of coughs or similar sounds) could serve as the first indication of a disease. Further exploring the nature of those symptoms would require the use of other pillars, most notably the *Attentively* one to determine whether the identified sounds represent a deviation from the “norm” of a given subject; this would serve as another indication of disease. In general, both systems would collaborate with the *Responsibly* pillar to take into account subject demographics. This would help contextualised detected patterns with respect to the particular risks faced by the individual.

This early screening system would mostly serve to provide warnings which trigger a subsequent medical evaluation. During a visit to a medical professional, the subject would present an account of their symptoms, which would be complemented by intelligent analytics from the auditory monitoring system. This highlights the need for the system to be explainable (a component of the *Responsibly* pillar), as merely reporting concerning findings without additional details or explanations about the nature of the detected pathology would be of little help to the practitioner.

Following a medical examination, the nature of which could also entail the use of computer audition technologies as well, the healthcare professional would prescribe an intervention (e.g. in the form of medication or surgery) or highlight potential causes of concern. The success of this intervention or the potential risks require subsequent monitoring, entrusted (partially) to a similar auditory monitoring system. This time, however, instead of a “broad sweep” for different comorbidities, the monitoring would be targeted to a particular disease, or at least a constrained set of alternative diagnoses prescribed by the practitioner. A diagnosis serves as a “primer” for all components of the *HEAR* system to look for a particular disease: the *Hear* component would be more sensitive to the biomarkers of choice, the *Earlier* pillar would draw on existing knowledge for those biomarkers to enhance their detection, *Attentively* would track changes according to the initial states, *Responsibly* would provide the missing link to patients and practitioners by interpreting those changes and transforming them to features understandable by both professionals and laymen.

In general, this second, more targeted phase would mean the beginning of a human-machine loop, where a medical practitioner prescribes interventions whose success the auditory system helps to quantify, or identifies missing information that the system needs to gather. Each time, a new prescription signals a new configuration of the *HEAR* system: *Hear* looks for the missing information, assisted by *Earlier* and *Attentively*, and their findings are reported back with the help of *Responsibly*.

Each pillar may encompass different capabilities across the two different operating modes. While all of them need to be active during the initial screening phase, with the system hearing with “all ears open,” there is an inherent trade-off in such a holistic approach: the more targets the system is looking for, the bigger the possibility of false alarms or confusions, and the greater the complexity of the overall system, with the accompanying increases in energy consumption and computational resources. This necessitates the presence of the second, more guided phase, where the system is looking for a more constrained set of biomarkers. In either case, there are stringent requirements for reliability and explainability that can only be satisfied with the use of prior knowledge, attention to the individual, and an adherence to ethical principles. Ultimately, it is user trust that is the deciding factor behind the adoption of a transformative technology. The use of computer audition in healthcare applications is currently in its nascent stages, with a vast potential for improvement. Our blueprint, *HEAR4Health*, incorporates the necessary design principles and pragmatic considerations that need to be accounted for by the next wave of research advances to turn audition into a cornerstone of future, digitised healthcare systems.

## Footnotes

^1^ https://icd.who.int/
